# Non-Coding RNAs and Immune Evasion in Human Gamma-Herpesviruses

**DOI:** 10.3390/v17071006

**Published:** 2025-07-17

**Authors:** Tablow S. Media, Laura Cano-Aroca, Takanobu Tagawa

**Affiliations:** 1 The Institute of Quantitative Biology, Biochemistry and Biotechnology (IQB3), School of Biological Sciences, The University of Edinburgh, Edinburgh EH9 3BF, UK; t.media@sms.ed.ac.uk (T.S.M.); laura.canoaroca@ed.ac.uk (L.C.-A.); 2 The Institute of Infection and Immunology Research (IIIR), School of Biological Sciences, The University of Edinburgh, Edinburgh EH9 3BF, UK

**Keywords:** Epstein–Barr virus, Kaposi’s sarcoma herpesvirus, immune evasion, non-coding RNA, oncogenesis, post-transcriptional regulation

## Abstract

Herpesviruses are DNA viruses that evade the immune response and persist as lifelong infections. Human gamma-herpesviruses Epstein–Barr virus (EBV) and Kaposi’s sarcoma herpesvirus (KSHV) are oncogenic; they can lead to cancer. Oncogenic viruses are responsible for 10–15% of human cancer development, which can have poor prognoses. Non-coding RNAs (ncRNAs) are RNAs that regulate gene expression without encoding proteins, and are being studied for their roles in viral immune evasion, infection, and oncogenesis. ncRNAs are classified by their size, and include long non-coding RNAs, microRNAs, and circular RNAs. EBV and KSHV manipulate host ncRNAs, and encode their own ncRNAs, regulating host processes and immune responses. Viral ncRNAs regulate host functions by post-transcriptionally modifying host RNAs, and by serving as mimics of other host RNAs, promoting immune evasion. ncRNAs in gamma-herpesvirus infection are also important for tumorigenesis, as dampening immune responses via ncRNAs can upregulate pro-tumorigenic pathways. Emerging topics such as RNA modifications, target-directed miRNA degradation, competing endogenous RNA networks, and lncRNA/circRNA–miRNA interactions provide new insights into ncRNA functions. This review compares ncRNAs and the mechanisms of viral immune evasion in EBV and KSHV, while also expanding on recent developments in the roles of ncRNAs in immune evasion, viral infection, and oncogenesis.

## 1. Introduction

Oncogenic viruses account for 10–15% of human cancers, some of which have high mortality rates [1,2]. Human gamma-herpesviruses are a subfamily of herpesviruses that are oncogenic—they are etiologic agents of various malignancies [3]. The two human herpesviruses that make up this category are Epstein–Barr virus (EBV) and Kaposi’s sarcoma herpesvirus (KSHV). EBV can lead to the development of Hodgkin lymphoma, Burkitt lymphoma (BL), nasopharyngeal carcinoma (NPC), and gastric carcinoma (GC, GaCa, EBVaGC). These cancers are responsible for over 200,000 cases a year, globally [4]. KSHV infection can lead to the development of malignancies such as Kaposi’s sarcoma (KS), primary effusion lymphoma (PEL), multicentric Castleman’s disease (MCD), or KSHV inflammatory cytokine syndrome (KICS). KS and PEL are cancers that typically occur in immunocompromised and/or AIDS patients, as an impaired immune system seen in these patients increases their susceptibility to infection and cancer development [5]. In 2020, there were 34,270 newly diagnosed KS cases, with 15,086 KS deaths reported worldwide. PEL has a survival rate of about 40%, highlighting the severity of oncogenic herpesviral infection and subsequent cancer development [6,7]. In terms of infection, KSHV and EBV primarily infect B cells, which are antigen-presenting cells important for the immune response against viral infection. KSHV also infects monocytes and macrophages, which affects differentiation and proliferation of these infected B cells. This persistent infection in leucocytes is what can lead to the aforementioned B cell malignancies seen with EBV and KSHV [8,9,10].

Herpesviruses have two stages of infection, lytic and latent, and different genes are expressed in each stage. While EBV-infected B cells become latent as memory B cells, EBV can also infect epithelial cells, which serve as primary sites of replication. During EBV latency, the viral genome is circularized and is tethered to host chromatin through use of viral protein EBNA1. Furthermore, EBV has three stages of latency (I, II, III), where each stage expresses different viral genes and proteins, and is linked to various EBV-associated diseases. Latency I is the most restrictive, expressing only a few latent viral genes, whereas latency III is the most immunogenic latency stage, expressing the full panel of latent viral genes and proteins [10]. For lytic infection and reactivation, Zta (also known as BZLF1, ZEBRA) and Rta (BRLF1) activate EBV early lytic genes, and viral DNA replication begins, triggering EBV lytic infection [11]. For KSHV, during latency, the viral genome subsists as a circularized episome tethered to host chromatin via latency-associated nuclear antigen (LANA) protein. Expression of transcriptional activator RTA induces KSHV lytic reactivation and lytic gene expression [12]. During the lytic stage, both EBV- and KSHV-infected cells produce viral factors that trigger the antiviral immune response in hosts. As a result, these viruses have evolved to use these lytic factors to their advantage to regulate the antiviral immune response [13,14]. This is observed as EBV and KSHV express lytic genes that target pattern recognition receptor (PRR) pathways and interferon signaling pathways [15]. PRR pathways recognize viruses through pathogen-associated molecular patterns, a mechanism essential for activating the innate immune response. For example, BPLF1 is an EBV lytic gene that can downregulate Toll-like receptor (TLR) activation. TLRs are a family of PRRs that interact with various proteins and transcription factors such as NF-κB to trigger the antiviral immune response [16]. Downregulation of TLRs subsequently downregulates NF-κB activation, highlighting how EBV uses BPLF1 to interfere with the innate immune response to infection [17].

As stated, KSHV and EBV primarily infecting B cells allows them to interfere with antigen presentation. This interference then dysregulates the adaptive immune response, leading to immune evasion. For example, EBV and KSHV impede major histocompatibility complex (MHC) class I and II presentation, proteins responsible for presenting foreign peptides to T cells to trigger the adaptive immune response [8,9,10,18,19,20]. Therefore, the ability of both EBV and KSHV to interfere with pattern recognition, interferon signaling, and antigen presentation demonstrates the importance of immune evasion mechanisms for the proliferation of these viruses.

KSHV and EBV further dysregulate innate immunity by affecting inflammatory pathways and cytokine signaling, promoting infection and developing their associated diseases. For example, KSHV induces cytokines to promote inflammation—a hallmark of KSHV pathogenesis. KSHV-associated diseases such as MCD and KICS have systemic inflammation as lytic KSHV infection leads to the manipulation of host cellular pathways, inducing host cytokines, dysregulating cytokine signaling [21]. KSHV infection in monocytes leads to the secretion of interleukins (ILs) IL-1α, IL-1β, and IL-6, all pro-inflammatory cytokines. Elevated levels of inflammatory cytokines like IL-6 and TNFα have also been reported in KS, favoring disease progression [22,23]. Elevated cytokine levels are also a hallmark of chronic active EBV infection. This disturbed cytokine profile in chronic active EBV infection favors EBV-infected cells and viral disease progression [24,25]. More specifically, the disruption of inflammatory cytokine signaling in EBV occurs via the release of IL-1 family member cytokines and their receptors, such as IL-1α and β, IL-18, and IL-33, by signaling pathways downstream of PRRs. Additionally, serum levels of IL-1α, IL-2, IL-6, and interferon gamma (IFN-γ) are reported to be very high in symptomatic patients with acute or chronic EBV infection. EBV also encodes its own IL-10 (vIL-10), which modulates inflammatory cytokine production and promotes B cell proliferation and differentiation, indicating the importance of cytokine dysregulation in EBV infection [25,26,27].

Furthermore, KSHV and EBV also evade immunity through the use of non-protein coding RNAs (ncRNAs). ncRNAs are not translated into proteins, but are still involved in a variety of cellular and immune processes [28]. They can be classified into three categories: (1) small RNAs (fewer than 50 nt); (2) RNA Polymerase III (Pol III) transcripts (tRNA, Y RNA), PolV transcripts in plants, small Pol II transcripts (snoRNA, snRNA); and (3) lncRNAs (greater than 500 nt) [29]. Some examples of ncRNAs are ribosomal RNA (rRNA), transfer RNAs (tRNAs), long non-coding RNAs (lncRNAs), microRNAs (miRNAs), stable intronic sequence RNAs (sisRNAs), and circular RNAs (circRNAs). These RNAs are being studied for their roles in cell differentiation, gene expression, cell signaling, and for their impact on innate and adaptive immunity [30,31]. KSHV and EBV manipulate host ncRNAs and encode their own ncRNAs to evade immunity and promote persistent infection.

The utilization of ncRNAs by herpesviruses is essential to infection; these RNAs can be expressed during latency, when few viral genes are expressed, allowing the viruses to regulate the host environment without inducing a large-scale immune response seen during lytic infection [32]. The ability of gamma-herpesviruses to evade immunity is significant because it leads to lifelong, persistent infection in hosts that can then lead to oncogenesis [33,34]. EBV and KSHV miRNAs can regulate host gene and miRNA expression to evade immunity.

This review intends to provide a thorough comparison of the ncRNAs and mechanisms the human gamma-herpesviruses KSHV and EBV utilize for viral immune evasion, infection, and oncogenesis. Recent developments in the emerging roles of ncRNAs in gamma-herpesvirus infection are also discussed, providing a dissection of the functions of EBV’s and KSHV’s viral ncRNAs, as well as their similarities and differences.

## 2. MicroRNAs

**Figure 1:**
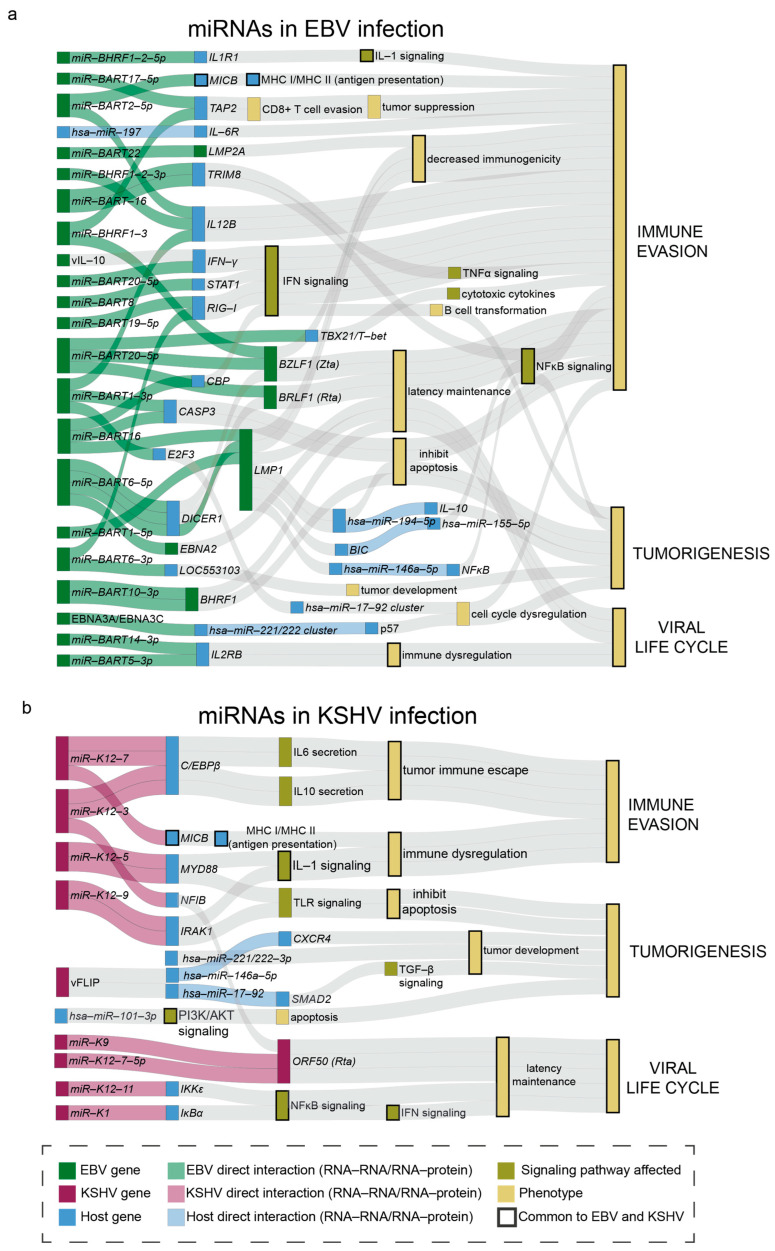
Overview of EBV and KSHV miRNAs regulating host and viral factors to promote immune evasion and oncogenesis. Schematic representation of the (**a**) EBV and (**b**) KSHV miRNAs and how the experimentally supported interactions regulate host factors to promote immune evasion and oncogenesis. Green: EBV factors. Blue: cellular factors. Yellow: the outcome of the miRNA regulatory mechanisms. Purple: KSHV factors. Direct interactions are highlighted in lighter colors. Bolded colors represent components/pathways impacted by both KSHV and EBV infection. *plotly* R-package was used for this visualization.

miRNAs are a group of small ncRNAs (~22 nt in length) capable of regulating gene expression, and as a result, many biological processes. Changes in miRNA expression are associated with a wide range of infections and can impact immunity. miRNAs also post-transcriptionally regulate mRNA levels in a targeted manner, regulating cellular processes. In this section, we review viral miRNAs, cellular miRNAs, extracellular miRNAs—and the recent developments regarding their roles in immune evasion during gamma-herpesviral infection (Figure 1).

### 2.1. Viral microRNAs

To date, over 1300 viral miRNAs (v-miRs) have been identified, the majority of which belong to the Herpesviridae family, highlighting the significance of viral miRNAs for herpesviral infection [35]. KSHV has 12 known miRNA precursors, producing 25 mature miRNAs [36,37,38,39,40] capable of regulating viral and cellular gene expression to promote viral infection [41,42,43]. The KSHV miRNA precursors are localized in the latency-associated region of the KSHV genome, where 10 of the precursors are found between the Kaposin and open reading frame 71 (ORF71) genes [38,44]. Alternatively, EBV is known to produce 25 miRNA precursors, containing 44 mature miRNAs. These miRNA precursors are also localized in the EBV genome; the BamHI fragment H rightward open reading frame 1 (BHRF1) gene encodes three miRNA precursors leading to four mature miRNAs, and the BamHI fragment A rightward transcript (BART) region contains twenty-two miRNA precursors that produce mature miRNAs [44,45,46].

Immune evasion is a key function of viral miRNAs encoded by EBV and KSHV. For example, ebv-miR-BART2-5p and kshv-miR-K12-7 decrease expression of major histocompatibility complex class I-related chain B (MICB) protein, which is important for infected B cells to activate T cells, and the antiviral immune response [47]. EBV v-miRs further interfere with antigen presentation, as EBV miRs BHRF1-3 and BART17-5p dysregulate MHC peptide loading, interfering with the TAP2 reporter, which is important for transporting peptides to CD8+ T cells to activate the adaptive immune response [48]. ebv-miR-BART1-3p and ebv-miR-BART2-5p also downregulate viral antigen presentation, leading to immune evasion [49]. ebv-miR-BART1-3p, ebv-miR-BART2-5p, and ebv-miR-BHRF1-2-3p target *IL12B*, which then restricts naïve CD4+ T cell differentiation, impairing the immune response [49]. Multiple groups have shown that ebv-miR-BART1-5p, -BART16, and -BART17 can suppress an EBV latent membrane protein, LMP1, in infected B cells and NPC, which inhibits the LMP1-mediated activation of co-stimulatory molecules and antigen presentation [49,50,51]. More recently, multiple EBV miRNAs like ebv-miR-BART3, 16 and 19 were found to suppress retinoic acid-inducible gene 1 protein (RIG-1) (DDX58) mRNA and the interferon (IFN) response, which are the first lines of immune defense [52,53]. ebv-miR-BART20-5p and ebv-miR-BART8 interfere with the IFN-γ-STAT1 signaling pathway, as higher levels of these miRNAs are inversely correlated with *STAT1* levels. miR-BART20-5p directly target transcripts of IFN-γ, and miR-BART8 directly inhibits *STAT1*. Decreased STAT1 reduces activation of the IFN-γ-STAT1 pathway, allowing for viral replication and tumor growth [54]. Furthermore, EBV miR-BHRF-1-2-5p downregulates signaling of the potent inflammatory cytokine interleukin-1 (IL-1) by targeting IL-1 receptor 1 (IL1R1), reducing the immune response to EBV infection, enhancing immune evasion [55]. Aside from seeing this in immune cells such as B cells, a similar regulation was observed in NPC and EBVaGC. ebv-miR-BART6-3p was found to inhibit the RIG-1 and IFN responses in NPC cell lines [56]. ebv-miR-BART5-5p directly inhibits a protein inhibitor of activated STAT 3 (*PIAS3*), which leads to the induction of an immune checkpoint molecule, PD-L1, in EBVaGC lines [57]. During KSHV infection, v-miRs such as kshv-miR-K12-5 and kshv-miR-K12-9 downregulate TLR signaling, impairing the innate immune response [58]. KSHV also targets the NF-κB pathway via kshv-miR-K12-1 and -11, dampening the immune response and promoting latency [59,60,61,62] (see Section 2.2 for more detail). Also, kshv-miR-K12-3 and kshv-miR-K12-7 induce IL-6 and IL-10 cytokine secretion from infected monocytes and macrophages, promoting tumor immune evasion [63].

Maintaining latency is a frequently hypothesized mechanism of immune evasion for both KSHV and EBV. Latent and lytic gene transcripts are regulated by EBV and KSHV ncRNAs, interfering with lytic reactivation, promoting latency, thus contributing to immune evasion, as viruses in latency produce less viral antigens than those in the lytic phase. Aside from enhancing immune evasion, the regulation of lytic and latent viral factors to control the replication cycle can also promote infection and oncogenesis. In KSHV, kshv-miR-K12-3 reduces the levels of nuclear factor I/B (NFIB), a transcription factor involved in RTA expression, decreasing lytic replication [64]. kshv-miR-K12-9 and kshv-kshv-miR-K12-7-5p directly suppress RTA expression, maintaining KSHV latency, evading immunity [65,66]. And kshv-miR-K12-1 inhibits lytic replication by interacting with RTA and NF-κB signaling to maintain latency [62]. More specifically, Lei et al. found that suppression of kshv-miR-K12-1 increases RTA and ORF57 transcripts. kshv-miR-K12-1 also binds to the 3’UTRof the transcript of the IκBα protein. This direct binding reduces expression of the IκBα protein, which is an inhibitor of the NF-κB complex, and the resulting activation of the NF-κB pathway can decrease KSHV viral lytic replication [62]. Alternatively, in EBV, latent ebv-miR-BART20-5p directly binds and inhibits transcripts of the immediate early (IE) transactivators BZLF1 (Zta) and BRLF1 (Rta), suppressing lytic EBV infection in a gastric carcinoma cell line, AGS [67]. And ebv-miR-BART6-5p downregulates DICER1 expression, which then decreases expression of the lytic reactivators Zta and Rta. ebv-miR-BART6-5p also suppresses EBNA2, a transcriptional regulator involved in the maintenance of latency. The suppression of EBNA2 may indicate a mechanism in which EBV switches from latency to promote infection [68]. Recently, ebv-miR-BHRF1-3 was found to directly interact with the BZLF1 transcript to suppress translation and the viral lytic cycle [69]. Overall, the extensive ways KSHV and EBV miRNAs regulate the immune response emphasizes the role of ncRNAs in immune evasion during EBV and KSHV infection.

### 2.2. Cellular miRNA and Mimicry by v-miRNA

Cellular miRNAs are important for antiviral immunity. However, EBV and KSHV have been observed to regulate some of these miRNAs and/or their targets to promote immune evasion, viral infection and oncogenesis.

EBV miRNAs have many cellular targets that are involved in immune responses. As a result, EBV regulates their expression to favor viral replication and immune evasion. For example, the aforementioned miR-BART20-5p targets TBX21/T-bet, a transcriptional activator of the IFN-γ pathway, and a regulator of IL-2 cytokine production. The inhibition of T-bet by ebv-miR-BART20-5p leads to reduced cytokine production, evasion of immunity, and increased invasiveness of EBV-positive lymphomas [70]. And ebv-miR-BART15-3p, which was previously mentioned, targets the NLRP3 inflammasome, inhibiting its activation and, therefore, pro-inflammatory cytokine production [71]. Furthermore, miR-BART16 and miR-BART1-3p target *CASP3*, which encodes Caspase 3, a key apoptotic mediator that, when reduced, leads to higher cell survival [72].

Infection-regulated cellular miRNAs have been found to control viral infection. hsa-miR-197 has been found to be upregulated in BL, which then downregulates expression of IL-6R, which is involved in oncogenesis, illustrating how upregulation of a cellular miRNA may benefit immune evasion by reducing oncogenesis [73]. In contrast, hsa-miR-194 is downregulated in EBV infection, increasing levels of IL-10, an anti-inflammatory cytokine, which promotes survival of EBV B lymphoma cells by reducing the inflammatory immune response [74]. Moreover, EBV infection induces miR-155, a host miRNA important in immunity and oncogenesis. In latency stages, EBV protein LMP1 induces miR-155’s RNA precursor, BIC [75]. Higher miR-155 levels then downregulate NF-κB signaling, leading to suppressed innate immunity [76]. EBV activating miR-155 expression is important in the immortalization process of EBV-infected B cells as it downregulates IFN responses, assisting in EBV immune evasion [76]. Thus, these mechanisms may indicate that miR-155 is induced to maintain latency and immune evasion.

**Figure 2:**
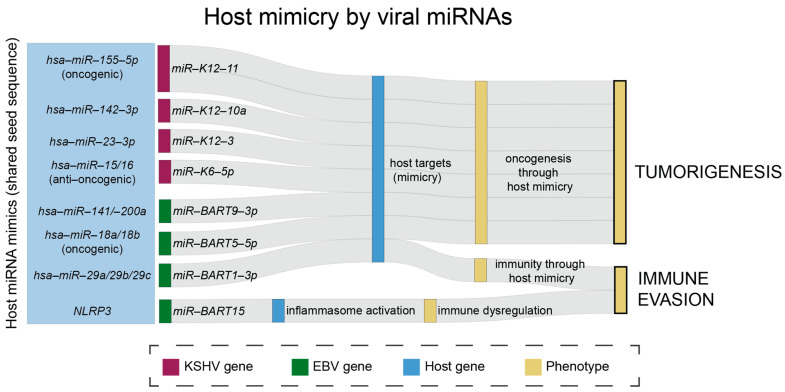
Overview of EBV and KSHV miRNA mimics regulating host factors to promote immune evasion and oncogenesis. Schematic representation of viral miRNAs that share seed sequences with host miRNAs, and how they may regulate host factors to promote immune evasion and oncogenesis. Green: EBV miRNAs. Purple: KSHV miRNAs. Blue: host miRNAs. Yellow: the outcome of the mimicry mechanisms. *plotly* R-package was used for this visualization.

KSHV also manipulates miR-155 expression, though in contrast to EBV, KSHV does this by encoding an miR-155 mimic, miR-K12-11 [60]. Viral mimicry is an important mechanism that EBV and KSHV both utilize to regulate cellular miRNA levels to their advantage. They do this by encoding miRNAs that have complementarity to the seed sequence of target mRNAs, the seed region being an important component for mRNA binding and for the gene-regulatory function of miRNAs [77]. miR-K12-11 shares the first 2–8 nt in its sequence starting at the 5’ end (seed sequence) with miR-155, and has been shown to regulate miR-155’s target genes, demonstrating how KSHV can mimic host miRNAs to promote viral persistence and oncogenesis, as high levels of miR-155 are associated with B cell development [60,61]. Other KSHV v-miRs, miR-K12-10a and miR-K12-3, share sequences with host miRNAs hsa-miR-142-3p and hsa-miR-23, respectively. Both of these v-miRs then act as cellular miRNA mimics to promote tumorigenesis [78,79]. KSHV miR-K12-6-5p can mimic the host anti-oncomiR miR-15/16 cluster, regulating their targets. Upregulating miR-K12-6-5p induces cell cycle arrest and tumor suppression, allowing KSHV to persist in hosts and evade immunity [80]. EBV miRNAs have also been found to share seed sequences with cellular miRNAs. In fact, the most abundant EBV miRNAs present during infection share seed sequences with cellular miRNAs. For example, EBV miRNAs that share seed sequences with cellular miRNAs include miR-BART9-3p, miR-BART5-5p, and miR-BART1-3p. The seed regions of these BARTs match with cellular miRNAs miR-141 and -200a [81,82], miR-18a/b [83], and miR-29a/b/c [84], respectively. miR-BART9-3p and hsa-miR-141 were found to share targets like *FOXO3*, promoting the viral lytic cycle [81]. In NPC, these ebv miRNAs that share seed sequences with host circRNAs are among most abundant, and corresponding host miRNAs are also dysregulated, though the mechanism is unclear. Anti-oncogenic miR-141/200a and miR-29a/b/c are downregulated, whereas miR-18a/b, which are part of the oncogenic miR-17-92 cluster, are upregulated in NPC [45]. The dysregulation of cellular miRNAs in NPC and the high abundance of EBV miRNAs with sequence homology to these cellular miRNAs suggest that these EBV miRNAs are highly abundant in order to mimic cellular miRNAs and control cellular function. Therefore, v-miR mimicry is a crucial mechanism EBV and KSHV both utilize to evade immunity and regulate the host immune response (Figure 2).

EBV and KSHV share some of the same cellular miRNA targets, but not many. And then these few shared targets can be up- or downregulated by EBV and KSHV, respectively. For example, miR-155 and miR-221/222 are upregulated in EBV oncogenesis, but downregulated in KSHV oncogenesis, potentially indicating different mechanisms of tumorigenesis for each virus [85,86,87]. Alternatively, miR-146a and miR-17 are upregulated during both EBV and KSHV infection, impairing immunity and promoting tumorigenesis [88,89,90,91]. While there are few identical cellular miRNAs induced in both EBV and KSHV infection, KSHV and EBV miRNAs commonly regulate similar pathways, such as antiviral immunity, lytic reactivation, and oncogenesis, as discussed earlier, partially because EBV and KSHV miRNAs share some cellular mRNA targets. For example, Gottwein et al. used PAR-CLIP, a method using Photoactivatable-Ribonucleoside-Enhanced Cross-linking and Immunoprecipitation to identify RNA binding protein (RBP) interaction sites on RNAs, along with Gene Ontology (GO) analysis, to determine whether specific targets of EBV and KSHV miRNAs are related to specific pathways. They then analyzed the overlap of these targets between the two viruses, concluding that fifty-eight percent of the 3’UTR targets of KSHV miRNAs had clusters with seed matches to EBV BART miRNAs, indicating many shared mRNA targets between EBV and KSHV miRNAs [78]. miRNA–mRNA targets are discussed further in Section 2.4.

The significant overlaps in mRNA targets between KSHV and EBV are especially noteworthy as there is no sequence conservation between their respective viral miRNAs [92]. Conservation of viral miRNAs is found within genera, i.e., the lymphocryptoviruses (EBV and rhesus lymphocryptovirus, rLCV) and rhadinoviruses (KSHV and rhesus rhadinovirus, RRV) [92]. The lack of conservation between EBV and KSHV miRNAs is important as it indicates convergent evolution of EBV and KSHV to target the same cellular pathways to evade immunity and promote infection. Though they lack conservation, KSHV and EBV miRNAs do share synteny: the miRNAs are located in their respective latent transcripts. The similarity in miRNA locations (synteny) in the EBV and KSHV genomes, despite the lack of sequence homology, strongly indicates a similarity between the pathways important to EBV and KSHV infection, evasion, and oncogenesis [93].

### 2.3. Extracellular Vesicle-Associated miRNA

miRNAs are not only found in the cell; they can be found in the extracellular space, packaged in extracellular vesicles (EVs), or as ribonucleoprotein complexes with proteins such as Argonaute (AGO) [94]. EVs are vesicles of cellular origin that play a role in intracellular communication. These vesicles and particles can carry a wide range of cargo from nucleic acids to proteins, mRNAs, cellular miRNAs, v-miRs, and other ncRNAs [95]. Therefore, there is an increasing amount of information regarding extracellular miRNAs on herpesviral infection and immune evasion. KSHV viral miRNAs have been found in EVs and play a role in altering neighboring cells. In one study of lymphatic endothelial cells (LECs), a Kaposi’s sarcoma model, Yogev et al. noted that KSHV miR-K12-10a-3p, K12-4-3p and K12-8-3p are present in higher levels in EVs, whereas KSHV miR-K12-11-3p and K12-4-5p are present in lower levels when comparing the amount of viral miRNA sequenced reads present in KSHV-infected LECs (K-LECs) versus their EVs. In K-LECs, viral miRNAs accounted for 5% of the total miRNA reads, whereas in exosomes secreted from K-LECs, viral miRNAs were about 10% of the total miRNA reads. These miRNAs in EVs are then able to be transferred to neighboring cells, and can potentially downregulate target genes [96]. Hoshina et al. reported that the human miRNAs miR-92a, miR-10b-5p, and miR-143-3p were highly abundant in EVs compared to cellular levels during KSHV infection, potentially indicating selective loading of these miRNAs to EVs for intercellular communication. They also found that viral miR-K12-3-5p was present in high levels in EVs in the KSHV BCBL-1 PEL cell line [97]. Chugh et al. evaluated the EV signature of KSHV and miRNA levels in KS patient plasma, pleural effusions, and mouse models of KS. They discovered that the oncogenic miR-17-92 and 106b/25 clusters are enriched in KSHV-derived EVs. Furthermore, they evaluated the functionality of KSHV exosomes and concluded that KS- and PEL-derived exosomes enhanced endothelial cell migration, which is significant for KS-associated angiogenesis [98]. Though miRNAs have been documented to be present in EVs during KSHV infection, functional regulation of immune genes through these EV miRNAs in KSHV infection has not been extensively established. The role of EVs must continue to be evaluated to fully understand their impact on viral infection and immune evasion.

EBV infection has similar outcomes, where miRNAs in EVs can be transferred to neighboring cells to regulate genes [96]. Some EBV viral miRNAs found in EVs so far include miR-BHRF1-3, miR-BART1-5p, miR-BART2-5p, and miR-BART3; these miRNAs are then internalized by recipient cells, suppressing target genes and the antiviral response [99]. Like KSHV, there is selective enrichment of certain EBV v-miRs in EVs when compared to cellular levels. This strengthens the case for the functionality of not only viral miRNAs but for that of EVs as well [99]. Furthermore, EV-miRNAs in EBV and KSHV infection continue to be characterized as regulators of host immune genes during infection. For example, BHRF1-3 miRNAs in EVs secreted by EBV-infected B cells reduce *CXCL11* gene expression in uninfected recipient cells, which promotes tumorigenesis [100].

However, the stoichiometry of EVs and associated miRNAs has been debated regarding whether EV-miRNAs are functional in recipient cells [101,102]. A previous evaluation of host miRNAs in EVs reported fewer than one molecule of a given abundant miRNA per exosome [101]. A recent study by Albanese et al. reported that EVs from EBV-infected cells do not contain many miRNAs, and that these EVs do not fuse with cellular membranes well enough to release cargo for functional purposes [102]. Furthermore, with the already few miRNAs they found in EVs, even fewer were transferred to recipient cells, and these transferred miRNAs did not appear to have functionality. They also demonstrated the importance of EV preparation methods and the need for the development of optimal EV functionality assays in EV-associated RNA studies [102]. These findings indicate that further evaluation of EV-miRNA content may be important in our understanding of EV function. EV purification and isolation is a challenge with EV studies; the field identifies extracellular vesicles and particles (EVPs) as a more coherent way to describe the particles isolated, as there is heterogeneity in EV isolations [103]. Within EVPs, there are what are known as exomeres and supermeres, which are small non-vesicular extracellular particles. Exomeres are smaller (<50 nm), non-membranous particles that co-isolate with EVs [104,105]. Exomeres contain mRNA and miRNAs, and supermeres have been noted to contain miRNAs as well. More specifically, exomeres contain the highest relative level of miRNAs in comparison to other EVPs [95,106]. As extracellular RNA research continues, EVP-associated RNAs have become important for viral immune evasion in KSHV and EBV infection. As KSHV and EBV ncRNAs have already been observed in EVs, further study will be imperative to understanding whether ncRNAs utilize EVs as a method to promote immune evasion, and also to determine if they are present in exomeres/supermeres.

### 2.4. miRNA–mRNA Network Discovery

miRNA–mRNA targeting is a mechanism that allows ncRNAs to evade immunity and promote infection. To understand miRNA–mRNA binding networks, we will first briefly discuss the biogenesis of miRNAs. miRNA formation occurs when primary miRNA (pri-miRNA) in the nucleus is transcribed and folded into a stem–loop hairpin to be cleaved by an RNase III enzyme (Drosha). The resulting precursor miRNA (pre-miRNA) is exported into the cytoplasm, and Dicer removes the loop, creating a ~22 base pair double-stranded RNA. This mature miRNA is loaded onto the effector protein AGO, forming the RNA-induced silencing complex (RISC). One miRNA remains in the RISC complex for mRNA targeting [107]. miRNA–mRNA binding in this complex refers to the binding of miRNAs to the 3’UTRs of target mRNAs as a post-transcriptional regulation mechanism. Watson–Crick base pairing (i.e., G·U, G·A, G·C) of the miRNA seed region to the target mRNA typically induces translational repression of the mRNA. As a result of miRNA–mRNA binding, the expression of target mRNAs is regulated by changes in expression of the particular miRNAs [108]. EBV and KSHV use miRNA–mRNA pairs to promote infection and evade immunity [78,84,109,110,111,112,113].

Argonaute-crosslinking and immunoprecipitation (AGO-CLIP), PAR-CLIP, Cross-Linking, Ligation and Sequencing of Hybrids (CLASH), and their respective variations are well-established methods that allow for the identification and characterization of AGO-binding and miRNA target sites across the transcriptome. CLIP identifies direct endogenous protein–RNA interactions by purifying short RNA fragments that crosslink to a specific protein, then identifying these fragments via sequencing [114,115]. CLASH allows high-throughput identification of sites of RNA–RNA interaction, identifying miRNA–mRNA interactions and characterizing the miRNA interactome [116]. These methods have allowed for the miRNA interactomes and miRNA targets of KSHV and EBV to be evaluated. As a result, the EBV and KSHV miRNAs and their mRNA targets have been characterized in a variety of cell lines. Thousands of miRNA targets have been identified, some of which have been validated experimentally. And pathway analyses indicate the processes the miRNA targets are involved in. Table 1 includes a few key CLIP/CLASH studies in EBV and/or KSHV infection, with their findings.

Briefly, in EBV, thousands of mRNA targets have been identified in Akata, Jijoye, SNU719, and LCLs for cellular and viral miRNAs [84,110,111]. These targets have then been implicated in a variety of pathways, including transcription, antigen processing and presentation, p53 feedback loops, B cell activation, apoptosis, and cell cycle control. Some of these targets have been experimentally validated, confirming their identity as targets and their proposed functions, seen with some BART miRNAs and miR-17 targets. For KSHV, thousands of mRNA targets have been identified in primary effusion lymphoma lines, BC-1 and BC-3, and in an endothelial cell line, TIVE-EX-LTC cells [78,112,113]. These targets were analyzed and found to be involved in transcription, intracellular signaling, apoptosis, and cell cycle control pathways. Validation of targets such as those of miR-155 and miR-K12-11 have demonstrated the value of CLIP/CLASH methods in successfully identifying miRNA–mRNA targets, allowing for the study of EBV and KSHV miRNA interactomes.

Furthermore, in an effort to uncover targets of EBV miRNAs, Ungerleider et al. conducted qCLASH, a comprehensive and quantitative approach of the Cross-linking, Ligation, and Sequencing of Hybrids method commonly used for identifying miRNAs and their targets [110]. They identified that the predominant targets of EBV miRNAs are transcripts of ubiquitin ligases and adapters, which are important for antiviral immunity. For example, ubiquitin ligase TRIM8, targeted by miR-BART-16, plays a key role in activating TNFα and NF-κB signaling pathways. The activation of these pathways leads to an increase in inflammation, promoting the immune dysregulation imperative for EBV infection. The consequence of miR-BART-16-TRIM8 being an miRNA–mRNA pair reduces antiviral immunity, indicating a mechanism for how EBV uses ncRNAs to evade immunity [110]. Additionally, EBV miRNAs ebv-miR-BART14-3p and ebv-miR-BART5-3p both target the interleukin 2 receptor subunit beta (IL2RB) transcript [117], which is important for regulating various interleukin pathways, leading to more severe EBV infection, as reduction in IL2RB interferes with T-cell mediated antiviral immunity [118]. These mRNA targets are also associated with the cellular senescence signaling pathway, so dampening these pathways will promote cell dysregulation [117].

miRNA–mRNA binding is crucial for EBV and KSHV to regulate host responses and evade immunity. Emerging ncRNA functions and miRNA–mRNA binding pairs continue to enhance our understanding of post-transcriptional regulation via ncRNAs. We have discussed the roles of miRNAs in KSHV and EBV immune evasion; how lncRNAs promote viral infection, immune evasion, and oncogenesis is discussed in the following sections.

## 3. Long Non-Coding RNAs

Long non-coding RNAs (lncRNAs) are a class of ncRNAs over 500 nucleotides in length that regulate gene expression by interacting with DNA, RNA, or proteins [29]. Virus-encoded and cellular lncRNAs regulate a variety of immune mechanisms, and KSHV and EBV manipulate lncRNAs to dampen the immune response to infection.

**Figure 3:**
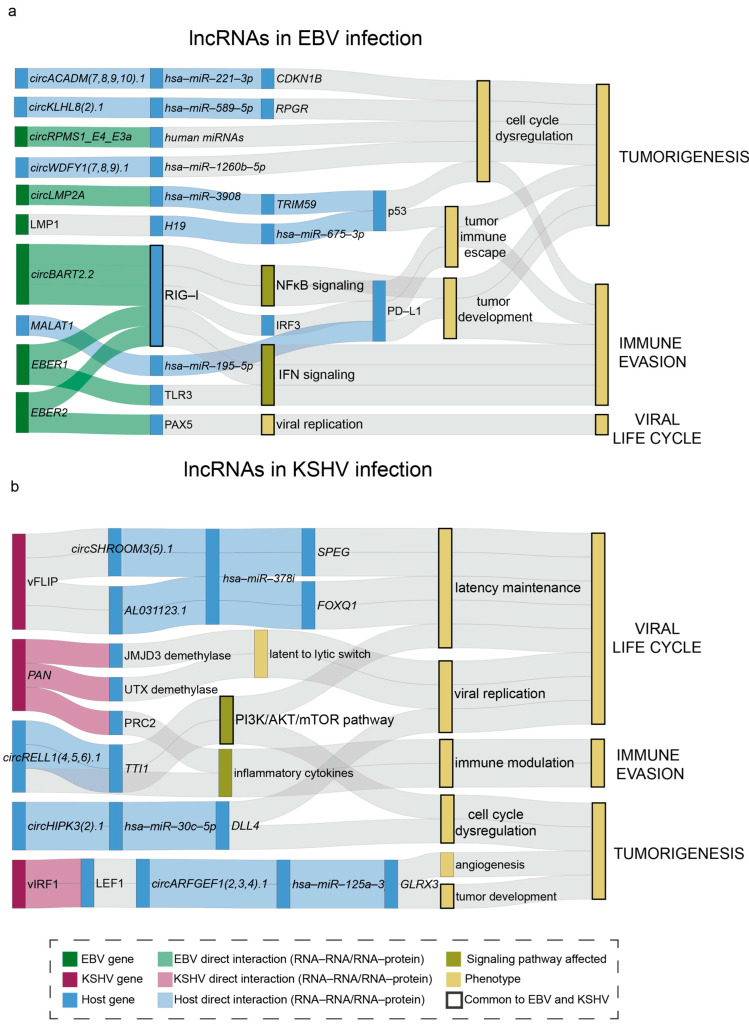
Overview of EBV and KSHV interactions with lncRNAs regulating host and viral factors. Schematic representation of the (**a**) EBV and (**b**) KSHV lncRNAs and how their interactions regulate host factors to promote immune evasion and oncogenesis. Green: EBV factors. Blue: cellular factors. Yellow: the outcome of the miRNA regulatory mechanisms. Purple: KSHV factors. Direct interactions are highlighted in lighter colors. Bolded colors are components/pathways impacted by both KSHV and EBV infection. *plotly* R-package was used for this visualization.

LncRNAs can post-transcriptionally regulate miRNAs by competing with other RNA transcripts for binding to the same miRNAs. In these interactions, lncRNAs are known as “competing endogenous RNAs,” or ceRNAs [119]. Research surrounding this method of miRNA regulation via ncRNAs has been expanding, and ceRNA “networks” are being mapped to understand the impact of these networks on regulation and immune evasion. LncRNAs can also post-transcriptionally regulate host processes by acting as miRNA “sponges,” sequestering and/or inhibiting miRNA function. In this section, we review lncRNAs important for KSHV and EBV infection, such as PAN, BARTs, BHRF1, and circRNAs, while also discussing lncRNA–miRNA interactions (Figure 3).

### 3.1. KSHV lncRNA: PAN

A viral ncRNA important to KSHV infection is long non-coding polyadenylated nuclear (PAN, also known as nut-1 or T1.1) RNA. PAN RNA is a polyadenylated RNA transcript transcribed by Pol II that localizes in the nucleus during lytic infection and plays a role in gene expression and immune modulation. PAN is the most abundant RNA transcript during lytic infection [120], and is quite stable; its stability is attributed to it containing an expression and nuclear retention element (ENE) at the 3′ end, which has a U-rich internal loop that hybridizes to and protects PAN RNA’s poly(A) tail. The U-rich loop of this ENE then forms a major-groove triple helix to interact with the poly(A) tail, further contributing to its stability [121]. Knockdown of PAN in KSHV-infected cells reduces viral gene expression, though the exact mechanism has not been established. It is thought that PAN RNA regulates gene expression via chromatin association [122,123]. PAN also interacts with interferon regulatory factor 4 (IRF4), and activates the interleukin-4 (IL-4) promoter. Furthermore, PAN decreases expression of inflammatory cytokines, such as IFN-γ and IL-18, interfering with the host immune response [123,124], highlighting its ability to enhance viral immune evasion.

### 3.2. EBV lncRNA: BARTs and BHLF1

In EBV, the aforementioned BART region responsible for EBV miRNAs also produces lncRNAs. The BART region in the EBV genome consists of a series of transcripts that share a 3’ terminus, and the BART RNAs are produced through alternative splicing. Interestingly, there are no large open reading frames (ORFs) the length of any of the identified BARTs. There are, however, smaller ORFs that may encode proteins. For example, the RPMS1 gene of the BART family is the only BART documented to have a full-length complementary DNA, and polymorphisms of the RPSM1 gene are being studied for their associations with EBV-associated malignancies [125]. Researchers have identified five major types, two of which were significantly associated with Hodgkin lymphoma and acute myeloid leukemia among a northern Chinese population, suggesting their roles in oncogenesis [125]. And, a notable small ORF spanning exons IV and V encodes for a RPMS1 isoform. The most abundant BARTs contain RPMS1 and BARF0 on the same transcript, which may encourage translation of RPMS1. However, the RPMS1 protein is not detected in EBV cell lines and tumors [126]. Nevertheless, the RPMS1 gene is associated with tumor development in NPC [127]. Whether the RPMS1 protein is encoded at detectable levels and the subsequent implications of this are still being evaluated [125]. Furthermore, knockdown of BART lncRNAs induces expression of genes coding for inflammatory cytokines such as IL6, interferons, and the interferon-stimulated genes (ISGs) OAS2, ISG20, IFIT2, and IFIT1 in an NPC line. BART lncRNAs stalled polymerase II on ISG promoters, suggesting that BART lncRNAs can regulate host gene expression through host chromatin remodeling machinery [128]. The mechanisms and other functions of lncBARTs are not well known, but are mainly being studied in the context of EBV-associated tumors such as NPCs, in which aberrant NF-κB signaling drives higher BART expression [129]. Recently, it was proposed that BART lncRNAs can act as sponges and interfere with host miRNA functions [130]. Investigating a gastric cancer cell line and correlating miRNA expression, miRNA target gene expression, and miRNAs complementarity to BART lncRNAs, researchers have proposed that a number of miRNAs such as let-7 are sequestered by the lncRNA to promote tumorigenesis [130].

Furthermore, BHLF1 is an early lytic EBV gene that has been noted to encode linear and circular ncRNAs [131,132]. BHLF1’s linear transcript has been implicated in promoting lytic replication of EBV [132]. And BHLF1 mRNA is mainly present as free RNA or associated with monosomal ribosomes. Regardless, BHLF1 was previously considered to be an actively transcribed gene, though there was no strong evidence to support this claim. Now, BHLF1 transcripts have been documented to be predominantly nuclear, and even latency-associated transcription of the BHLF1 locus has been proposed. As a result, BHLF1 transcripts may serve as lncRNAs and function as mRNAs for BHLF1 protein expression, indicating roles of BHLF1 lncRNA in B cell transformation during EBV infection [133].

### 3.3. Circular RNAs (circRNAs)

Circular RNAs are lncRNAs with covalently linked 3’ and 5’ ends, resulting in closed, circular structures. The discovery of circular RNAs occurred in the 1970s, but only in the last decade or two have circRNAs been characterized for functionality, especially in terms of immunity and oncogenesis [134,135]. circRNAs are currently divided into four classes, depending on their biogenesis: exonic circRNA (ecircRNA), circular intronic RNA (ciRNA), exon-intron circRNAs (EIcircRNA), and intergenic circRNAs [136]. circRNAs have their canonically spliced linear counterparts, whose mRNAs usually code for a variety of different proteins. circRNAs are typically expressed in lower levels than the linear RNA isoform, but there are instances where circRNAs can be present in higher levels than the linear RNAs. In terms of stability, circRNAs’ closed structure protects them from exonuclease degradation, contributing to their considerable stability—circRNAs can have a half-life of over 48 h in cells [136].

Furthermore, circRNAs are being studied for their roles in gene regulation, transcription, and miRNA sponging. In terms of immunity, circRNAs have been found to be involved in the regulation of immune cells and immune responses. Interestingly, circRNAs are noted to be decreased upon viral infection with a negative-strand RNA virus, Vesicular stomatitis Indiana virus, due to the cytoplasmic transport of RNA-binding proteins NF90/NF100 [137]. NF90/NF100 normally promote circRNA biogenesis in the nucleus, and form complexes with circRNAs, but when released from circRNAs upon infection to the cytoplasm, they target viral RNAs and suppress replication. Ectopic circRNA expression indeed facilitates viral infection and replication, highlighting circRNAs’ potential effects on immunity [137]. Furthermore, circRNAs can preferentially bind to PKR, acting as endogenous PKR inhibitors. PKR is a PRR that binds dsRNA. dsRNA is a common feature of many viruses and elicits an immune response when detected by PKR in hosts. Therefore, the ability of circRNAs to bind PKR indicates a mechanism in which circRNAs can interfere with and evade innate immunity [138]. Additionally, how the immune response differentiates between foreign and endogenous circRNAs is still being evaluated. Endogenously produced circRNAs have been reported to not activate the antiviral immune response, unlike exogenously produced circRNAs [139]. However, unmodified exogenous circRNAs have also been found to evade immune activation, evading recognition from TLRs and RIG-1 [140]. circRNAs modified with N6-methyladenosine are also able to evade immune activation, as RNA modifications are utilized by endogenous circRNAs to be recognized as endogenous, and not activate the immune response [141]. Therefore, circRNAs are able to evade immune activation, making them ideal tools for viruses to promote infection and evade immunity.

Mechanistically, circRNAs are lncRNAs that exhibit miRNA sponging capabilities, especially in ceRNA networks. Unlike traditional miRNA sponging, in ceRNA networks, lncRNAs can bind to miRNAs and inhibit the traditional miRNA-targeted degradation of mRNAs, increasing mRNA levels. Novel circRNA–miRNA interactions in infected cells are emerging; the roles of circRNAs in KSHV and EBV infection are outlined below.

The EBV BART loci encode circular RNAs. The RPMS1-derived circRNAs are from the RPMS1 BART locus. Ungerleider et al. and Toptan et al. identified EBV viral circRNAs with 16 variants [131] from the RPMS1 region [142,143]. Ectopic expression of circRPMS1_E4_E3a in a gastric cancer cell line increased cell migration rate [142], and downregulated 11 of 14 human miRNAs identified as potential targets of circRPMS1_E4_E3a via bioinformatic analysis predicting human miRNA binding sites of circRPMS1_E4_E3a [142]. Knockdown of circRPMS1 in NPC lines, in turn, decreased cell growth in vitro and tumor volume in a nude mice model, further corroborating the circRNAs’ oncogenic functions [144]. Inhibitors of host miRNAs miR-200, -31, and -203, reversed this phenotype, suggesting circRPMS1s serve as sponges of anti-oncogenic miRNAs [144]. Furthermore, BHLF1 encodes circBHLF1, which has been found to be expressed highly during the lytic stage of EBV infection. circBHLF1 also has an alternatively backspliced isoform, circBHLF1-alt, formed by a non-canonical splice donor upstream from the circBHLF1 splice donor. circBHLF1-alt was found to be present at higher levels than circBHLF1 in reactivated Akata cells. Both isoforms localize in the nucleus during EBV reactivation; their roles in EBV infection have not yet been defined [131].

KSHV also encodes viral circRNAs with various functions. Most KSHV circRNAs are encoded by viral interferon regulatory factor 4 (vIRF4) and PAN. For example, circvIRF4 and circPAN/K7.3 are two abundant KSHV circRNAs with potential regulatory roles that require further study [143,145]. Most recently, a study was published characterizing potential functions of circvIRF4. Torres et al., 2025 discovered that many genes are differentially expressed during latency and reactivation with circvIRF4 [146]. Restricting circvIRF4 formation impacted pathways related to cell adhesion, migration, and proliferation, as well as PI3K-Akt, JAK-STAT, MAPK, NLR, and IL-17 signaling pathways. However, it is important to note that the production of circRNA from the vIRF4 region was not completely eliminated in this study, as alternative backsplice donor sites were utilized, yielding novel isoforms of circvIRF4. As a result, the observed phenotypes and differential gene expression may not be directly tied to circvIRF4 abrogation. More research is needed to identify the exact mechanisms in which circvIRF4 may regulate gene expression [146]. Aside from KSHV-encoded circRNAs, human circRNAs are dysregulated in KSHV infection. For example, KSHV upregulates host’s circHIPK3(2).1, which sponges miR-29b and miR-30c, miRs that inhibit KSHV viral replication [147]. Viral interferon regulatory factor 1 (vIRF1) encoded by KSHV increases the transcription of circARFGEF1(2,3,4).1, which targets miR-125a-3p, preventing degradation of GLRX3 by the miRNA. This results in increased cell migration, proliferation and angiogenesis [148]. Human circRNA circRELL1(4,5,6).1 is induced by EBV, KSHV, and human cytomegalovirus (HCMV); circRELL1(4,5,6).1 serves pro-growth and anti-lytic replication functions in KSHV infection. This mechanism is hypothesized to be a tactic of KSHV to maintain latency and evade immunity. As circRELL1(4,5,6).1 is also induced by EBV and HCMV, regulation of this circRNA may result in similar phenotypes in these infections [145,149].

In certain cancers, circRNA expression levels may contribute to tumor development and immune dysregulation. For example, BART encoded EBV circRNA, circBART2.2, binds RIG-1 and activates the transcription factors interferon regulatory factor 3 (IRF3) and NF-κB, exacerbating the inflammatory immune response to promote tumor escape [150]. Tumor immune evasion is supported by circRNA regulation of various immune checkpoints, most notably the PD-L1 pathway, which is important for T cell activation, proliferation, and cytokine production. For example, the aforementioned circBART2.2 upregulates PD-L1 expression in NPC, leading to tumor escape. And circLMP2A is an EBV circRNA encoded by the LMP2A gene; circLMP2A can sponge host miR-3908, allowing for an E3 ligase, the miRNA’s target, to degrade p53, the tumor suppressor, promoting oncogenesis [151]. Furthermore, NPC shows very different expression patterns of host circRNAs. For example, circACADM(7,8,9,10).1, circKLHL8(2).1, and circWDFY1(7,8,9).1 are overexpressed in EBV-positive cell lines. circACADM(7,8,9,10).1 targets miR-221-3p/CDKN1B, increasing cell cycle progression from G1 to S. And circMDM2(6,7,8).1 targets miR-589-5p, which may promote NPC development [152]. For KSHV, infection-induced circRELL1(4,5,6).1 interacts with and stabilizes *TTI1*, a transcript that codes an mTOR complex component. The AKT1/mTOR pathway is important for cell growth and proliferation, and the dysregulation of this pathway is often found in tumor development, including KSHV-driven oncogenesis and latency maintenance [149].

The field of circRNA research is still being expanded. circRNAs have been found in EVs, and are being analyzed for functional roles, both intra- and extracellularly. circRNAs being loaded into EVs may indicate a mechanism for clearing these circRNAs from cells [153]. Additionally, recent efforts in circRNA sequencing and identification have led to the use of circRNAs as biomarkers in disease. The dysregulation of circRNA expression levels in cancer makes them useful as biomarkers for diagnosis. circRNAs are also being studied in vaccine development, as they have similar qualities to mRNA in terms of protein translation, and yet they do not degrade as quickly as mRNA, meaning circRNAs are able to produce proteins for longer periods [154]. As circRNAs continue to be characterized, their effects on immune evasion of EBV and KSHV also continues to develop.

### 3.4. Post-Transcriptional Regulation of miRNAs via lncRNAs

A mechanism of lncRNAs recently being studied are lncRNA–miRNA interactions. lncRNA post-transcriptional regulation of miRNAs is carried out via miRNA sponging, or by the lncRNAs serving as miRNA precursors, or acting as binding competitors [119]. Also, target RNA-directed microRNA degradation (TDMD) is being characterized as an emerging technique of miRNA regulation via lncRNAs.

miRNA sponging is the most well-known mechanism of post-transcriptional regulation of miRNAs employed by lncRNAs. For example, long intergenic non-coding RNAs (lincRNAs) are a type of lncRNA; they make up around 50% of lncRNAs. LincRNAs do not overlap annotated coding genes, meaning they are not found on or within any coding genes. *MALAT1* is a lincRNA studied extensively in the context of EBV and KSHV. *MALAT1* is upregulated during KSHV tumor development to promote oncogenesis [155]. Furthermore, *MALAT1* is upregulated in the EBV-associated cancers NPC and diffuse large B cell lymphoma (DLBCL), promoting tumorigenesis [156,157]. MALAT1 was found to sponge miR-195 and increase PD-L1 expression in DLBCL patient samples and a derived cell line, promoting tumor proliferation and immune escape [157]. Therefore, it is possible that MALAT1 in EBV-associated DLBCL may also regulate oncogenesis as an miRNA sponge. Alternatively, for miRNAs regulating lncRNAs, ebv-miR-BART6-3p inhibits cancer cell migration by downregulating lncRNA-LOC553103 [158]. In KSHV, viral miRNAs are also being studied for their ability to downregulate host lncRNAs to regulate gene expression [159]. Researchers determined that cancer-associated lncRNAs were dysregulated by KSHV in v-miR-dependent (tumor suppressor *MEG* and oncogenic *ANRIL*) and independent (oncogenic *ANRIL* and *UCA1*) manners in endothelial cell lines [159].

Additionally, lncRNAs can serve as miRNA precursors (pre-miRNAs). These lncRNAs can be cleaved to form mature miRNAs that can serve various functions and play a role in gene regulation. Many lncRNAs that serve as miRNA sponges also serve as miRNA precursors. For example, lncRNA-H19 functions as a miRNA sponge for the let-7 family of miRNAs, which play a role in oncogenesis [160,161]. lncRNA-H19 is also a miRNA precursor that produces miR-675-5p and miR-675-3p [162]. LMP1 encoded by EBV downregulates lncRNA-H19, reducing miR-675-5p expression, leading to p53 overexpression in tumor cells of certain EBV-associated cancers [163].

Furthermore, ceRNA networks are similar to the miRNA–mRNA binding pairs outlined in Section 2.4. These networks involve a lncRNA that regulates an miRNA, which then impacts mRNA target levels. For example, KSHV vFLIP is a latent protein that is known to suppress viral reactivation. Recently, vFLIP was found to upregulate two ceRNA pathways, the circRNA circSHROOM3(5).1/hsa-miR-378i/SPEG/FOXQ1, and the lncRNA AL031123.1/hsa-miR-378i/SPEG/FOXQ1 axes, leading to an increase in mRNAs *SPEG* and *FOXQ1*. Both transcript levels increased after viral reactivation, suggesting a potential role of these networks in KSHV reactivation, though the exact mechanism has yet to be evaluated [164].

As an alternative to traditional miRNA–mRNA binding, binding of the entire length of the miRNA, not just the seed region, leads to siRNA-like endonucleolytic cleavage of the mRNA, which is important in TDMD. TDMD occurs when RNA binds not only to the 3’ end of the host miRNA, but continues with complementarity through to the seed region as well. This leads to active degradation of miRNAs, rather than a decrease in transcription or inhibition of pre-miRNA processing [165,166]. Furthermore, herpesviral ncRNAs have been found to be involved in TDMD, as seen in Herpesvirus saimiri and cytomegaloviruses in which abundant viral transcripts degrade specific host miRNAs [167,168,169]. As a similar but novel phenomenon, in human herpesvirus 6A, viral miRNA miR-aU14 was found to interfere with the maturation process of host miRNA miR-30, resulting in the disruption of mitochondrial architecture [170]. In relation to KSHV and EBV, ncRNAs are still being evaluated for their role in TDMD. For example, the aforementioned ebv-circLMP2A has been found to sponge miR-3908. ebv-circLMP2A has three predicted binding sites for miR-3908, potentially indicating that ebv-circLMP2A may inhibit miR-3908 via TDMD [151]. Additionally, the BART lncRNAs share sequence homology with many cellular miRNAs, often sharing multiple sites of homology, strengthening the hypothesis that BART lncRNAs engage in TDMD. BART lncRNAs target miRs whose targets inhibit tumor invasion, metastasis, and epithelial mesenchymal transition, potentially via TDMD [130]. For KSHV, circHIPK3(2).1, as previously mentioned, also sponges miR-30c and has been proposed to do so via TDMD, though further analysis is required to support this hypothesis [147]. Furthermore, a recent study retargeted known TDMD pairing sites to KSHV miR-K12-11, miR-K12-3, miR-K12-1 and miR-K12-4-3p, as well as human miR-122 and miR-155. They found that retargeting one of these sites to these miRNAs led to TDMD of the miRNAs. These findings illustrate the potential of using natural miRNA pairings to enhance TDMD performance, as well as for predicting seed matching to study TDMD of predicted targets [171]. While more study is needed to uncover TDMD capabilities of KSHV and EBV ncRNAs, TDMD is a conserved regulatory mechanism important for viral regulation of host miRNAs.

## 4. Other ncRNAs

### 4.1. EBERs

EBV-encoded RNAs (EBERs) are highly expressed ncRNAs during EBV infection. Also known as intermediate ncRNAs, EBERs are 167nt in length, falling into category two of the previously defined ncRNA classifications. EBER1 and EBER2 are present in all latency stages of EBV and interact with host RNA-binding proteins to promote infection [172].

EBERs are non-polyadenylated and transcribed by RNA polymerase III. EBER1 folds into four stem loops, and each loop binds a ribosomal protein. EBER2 has a single stem–loop structure and is typically in the terminal repeat (TR) region, potentially serving as a lytic replication regulator [172,173]. Furthermore, EBERs can interact with the host innate immune response to promote oncogenesis by activating RIG-I overexpression that contributes to EBV oncogenesis [174,175]. And EBER1 forms a complex with the cellular lupus erythematosus-associated antigen (La) protein, allowing EBER1 to be secreted from EBV-infected cells and interact with TLR3, which activates inflammatory cytokines, ultimately leading to immune activation via a TLR3-dependent signaling pathway [176]. EBER1 has also been found to induce IL-10 secretion, promoting BL development [177]. EBER2 has been shown to interact with B cell transcription factor PAX5, an interaction mediated by EBER2 and nascent transcripts from the TR locus base-pairing, a previously undiscovered capability of ncRNA. EBER2 knockdown decreases EBV lytic infection, highlighting the importance of EBER2 localizing PAX5 to the TRs of the latent EBV genome [178]. Interestingly, as EBV infection decreases TLR7 and TLR9 signaling, Bouvet et al. analyzed the impact of EBERs in this interaction and their role in IFN signaling. The EBERs do not directly regulate TLR7 or TLR9, nor do they regulate the type I IFN response to EBV infection, unlike the EBV miRNAs miR-BART19-5p and miR-BART22 [53]. EBERs are seen to typically exacerbate the host immune response, rather than evade it, making them important ncRNAs for EBV to promote infection and oncogenesis.

### 4.2. sisRNAs

Stable intronic sequence RNAs (sisRNAs) are an emerging group of ncRNAs around ~300 nt in length, and are typically expressed from genes with multiple introns, often derived from GC- and TG-rich regions of the introns [179]. These RNAs are stable, with 5’ caps and a 3’ poly(A) tail, and are found in a wide range of organisms, including yeast, humans, and viruses. In humans, sisRNAs mainly localize in the nucleus [180]. A noteworthy class of circRNAs in this category is EIcircRNAs, which are formed via backsplicing, and contain introns and exons [181]. The functions of sisRNAs are still being evaluated, but they are considered to play a role in the regulation of cellular processes by serving as protein or miRNA decoys for lncRNAs and other circRNAs (Section 3). EIcircRNAs have been found to regulate gene transcription in the nucleus [181]. sisRNAs present in oncoviruses have not been extensively studied, though sisRNAs have been identified in EBV. EBV has been found to encode two sisRNAs: ebv-sisRNA-1, and ebv-sisRNA-2. ebv-sisRNA-1 and ebv-sisRNA-2 originate from introns in the BamHI W repeat region, more specifically, from the introns around W1 and W2, which are exons that encode functionally active repeat domains of EBNA-LP [182]. Furthermore, intronic mutations in the EBNA-LP gene have led to reduced transformation ability of B cells, demonstrating the potential importance of ebv-sisRNA-1 during EBV infection [183]. ebv-sisRNA1 is present in high copy numbers and localizes in the nucleus [184]. ebv-sisRNA-1 and -2 can be bound by host regulatory proteins, such as HuR, hnRNP A1/C/DL and Lin28, which are involved in regulating host gene expression, also indicating the functionality of these sisRNAs, though this must continue to be evaluated [182]. While information on sisRNAs is limited, these ncRNAs appear to be valuable regulatory molecules, and further evaluation in oncoviruses can determine their roles in evasion and tumorigenesis.

### 4.3. TMERs

Murine Gammaherpesvirus 68 (MHV68) is a small-animal model gamma-herpesvirus that leads to oncogenesis. MHV68 encodes transfer RNA (tRNA)-miRNA encoding RNA molecules, or TMERs [185]. They are expressed in latency and are similar in size and structure to EBERs. TMERs have a tRNA-like structure at the 5’end, and hairpins processed into miRNAs which can post-transcriptionally regulate other RNAs [186,187]. TMERs appear to contribute to pathogenesis by interacting with host proteins. For example, TMER4 is a TMER in MHV68 that has been shown to be important for dissemination of infected cells to latent sites, and has been reported to play a role in promoting infected cell survival [188]. Recently, TMER4 was studied for potential immunomodulatory roles; Kara and Tibbetts determined that TMER4 did not alter TLR or RIG-1 signaling, unlike the EBERs [189]. TMER4 was further analyzed to see if it shared any similarity with the EBER molecules in EBV. TMER4 and EBER1 do not share a secondary structure, though they are similar in terms of size and expression levels during latency. Interestingly, Hoffman et al. conducted a study where TMER4 was replaced with EBER1 in mice, which was able to restore phenotypes affected by TMER4 removal such as viral establishment of latency, strongly indicating conserved function between EBER1 and TMER4 [190]. TMERs and MHV68 may not be directly relevant to human herpesviruses due to their lack of sequence conservation, but, as shown for EBER1, MHV68 can serve as an in vivo model to study human gamma-herpesviruses and ncRNAs, especially considering human gamma-herpesviruses lack an animal model.

## 5. RNA Modifications

RNA modifications are a form of post-transcriptional regulation involving the addition or removal of chemical groups, expanding the variety of roles RNAs can have in immune regulation and disease progression. In the past few years, different types of RNA modifications have been found in various ncRNAs, including viral ncRNAs. The impacts of these modifications on viral infection and immune regulation are still being elucidated.

The N^6^-Methyladenosine (m6A) RNA modification is an extensively studied RNA modification that can have pro-viral or antiviral functions in various viral infections [191]. For EBV, m6A RNA modifications influence latent and lytic replication. m6A modifications of viral transcripts are higher during latency stages of EBV infection, potentially indicating a mechanism related to how EBV maintains long-term infection and cancer development in hosts. m6A modifications have been found on many EBV transcripts, including BZLF1 and BRLF1, destabilizing them and promoting latency [192]. Furthermore, EBV infection upregulates METTL14, a component of the m6A methyltransferase complex, to promote tumorigenesis. Upregulation of METTL14 maintains EBV latent infection as well, leading to EBV evading immunity and persisting in hosts [193]. The m6A modification of the *TLR9* transcript is inhibited by EBV infection, reducing its stability, allowing EBV to evade immune recognition by TLR9 [194]. METTL14 upregulation promotes growth and proliferation of EBV-infected cells, and knockdown of METTL14 resulted in a decreased tumorigenic activity of EBV-infected cells. Alternatively, EBV can interfere with the m6A modification system during the lytic stage of EBV infection and reactivation to promote EBV infection [195]. EBV downregulates the expression level of the m6A eraser ALKBH5 during reactivation, and this downregulation impairs the IFN response, allowing EBV to subvert immunity [196]. In relation to RNA modifications of EBV ncRNAs, EBER1 displays a conserved 5-methylcytosine (m^5^C) modification at C145 located within stem–loop 5, deposited by NSUN2 RNA methyltransferase. This EBER1 modification is targeted by angiogenin RNase in vivo, leading to decreased levels of this ncRNA, potentially reducing EBV recognition via PRRs, as EBER1 is immunogenic [197]. In EBER2, a conserved Ψ114 site was found across different cell lines, the significance of which is still being evaluated [198].

KSHV has also been observed to use m6A modifications to suppress or activate lytic replication, both serving different viral advantages. Ye et al. determined that most KSHV-encoded transcripts undergo m6A modification, which promotes lytic gene expression, as the blocking of m6A modifications reduced lytic gene expression and virion production. During KSHV lytic replication, m6A-modified viral transcripts are increased, leading to lytic gene expression and viral replication [199]. However, m6A modifications can also have the opposite effect. For example, YTHDF2, an m6A reader protein that mediates mRNA degradation, has been found to suppress KSHV lytic replication by degrading viral methylated lytic transcripts [200], a cellular mechanism that favors KSHV latency. Furthermore, during lytic reactivation, KSHV utilizes the RNA-specific adenosine deaminase 1, which mediates adenine-to-inosine (A-to-I) editing in mRNAs, to dampen RIG-1-like receptor (RLR) signaling and the innate antiviral immune response, promoting infection [201]. Comparison of KSHV A-to-I editomes in different PEL cell lines revealed common edits in miRNAs and ncRNAs. For example, three conserved A-to-I modifications of the pri-miRNA-K12-4 transcript were found to alter miRNA biogenesis and target specificity. Additionally, editing of the seed sequence of pri-miRNA-K12-4 is necessary for high levels of KSHV infection, highlighting the significance of this RNA modification for KSHV infection [202]. And KSHV PAN stability and viral replication are strengthened by N4-acetylcytidine (ac4C) modifications [203]. RNA modifications are an emerging field, and as modifications continue to be observed in host and viral ncRNAs, characterizing their roles will reveal their impact on viral infection and cancer progression.

## 6. Conclusions

The field of ncRNAs is an ever-growing area of research (Table 2). In the context of the gamma-herpesviruses EBV and KSHV, ncRNAs are imperative for viral immune evasion, promoting lifelong infection and the development of virus-associated cancers. EBV and KSHV can upregulate or downregulate host ncRNAs, regulating immune pathways to promote viral infection and oncogenesis. These viruses also encode their own ncRNAs, inhibiting host miRNA functions to further infection and evade immunity. As ncRNAs continue to be characterized, previously unevaluated lncRNAs like circRNAs are being recognized for their functions against and interactions with host immunity. With this, the construction of ceRNA networks and their impact on infection and evasion is emerging. EVs are also being implicated in ncRNA functionality, with the delivery of ncRNAs via EVs influencing the surrounding cellular environment. RNA modifications in ncRNA are also being evaluated, and appear to influence ncRNA function to promote infection. And the presence of extracellular RNA has been noted in the cytoplasm, the implications of which are in the preliminary stages of study. Our understanding of ncRNAs continues to grow: exactly how “non-coding” they are is beginning to come into question. A recent study has demonstrated that lncRNAs have short open reading frames (sORFs) that produce micropeptides. These micropeptides are then bound by MHC molecules, allowing the host to activate an adaptive immune response [204,205]. As micropeptides receive more attention, and the development of the immunopeptidome and influence of sORFs are characterized, it may emerge that lncRNAs have more traditional coding roles with their regulatory effects, similar to other RNAs. More tools and ways to study viral ncRNAs are imperative to further our understanding regarding these ncRNAs. Greater depth of RNA sequencing has allowed for stronger profiling of ncRNAs. Being able to identify ncRNAs in different cellular conditions can then allow for their subsequent targeting in functional assays. And analyzing lncRNA–protein interactions at high resolution using emerging modeling techniques can only serve to enhance our understanding of the significance of lncRNAs [206]. Therapeutically speaking, ncRNA expression is altered during oncogenesis, and therefore, ncRNAs are being utilized as biomarkers for cancer development, as well as biomarkers for predicting patient survival in certain cancers. And these ncRNAs are ideal drug candidates; targeting them with antagonists can reduce their pro-viral and pro-oncogenic effects. Furthermore, as circular RNAs can be expressed endogenously and have longer half-lives than mRNA, they are being evaluated in the context of vaccine development [207]. Research on the function of ncRNAs, originally thought to be waste, now known to be immune modulators and micropeptide encoders, is crucial to better understanding mechanisms of viral infection and evasion, to eventually leading to better management of herpesviral infection and oncogenesis.

## Figures and Tables

**Table 1:** CLIP/CLASH studies on EBV and KSHV.

Virus and Cell Line	Method	miRNA–mRNA Targets Identified	Validated Targets	Pathway Analysis—Top Pathways	Findings	Ref
KSHV (BC-1, BC-3)	PAR-CLIP	Identified 1741 and 1409 target mRNAs in BC-1 and BC-3, respectively. KSHV miRNAs directly target more than 2000 cellular mRNAs.	Confirmed identified targets of miR-155. Confirmed 12 out of 29 KSHV miRNA targets, with expression >40%.	Regulation of transcription, intracellular signaling cascade, and protein localization.	It was found that 58% of mRNAs targeted by KSHV are also targeted by EBV miRNAs. KSHV encodes a viral miRNA that mimics cellular miR-142-3p function.	[78]
KSHV (BC-1, BC-3)	HITS-CLIP	Identified 1170 and 950 cellular KSHV miRNA targets from BCBL-1 and BC-3 cells.	Confirmed 10 of 12 miR-K11 targets. Validated vIL-6 as a miR-K12-10 target.	Apoptosis, glycolysis, and lymphocyte activation.	Only had 42% overlap with Gottwein method.	[112]
EBV (Jijoye)	HITS-CLIP	mRNA targets of 44 EBV and 310 human miRNAs.	LMP1 repression via BART miRNAs and host miRs. miR-17 family targets were validated.	Transcription, apoptosis, Wnt signaling, and the cell cycle.	miRNAs do not control the latent/lytic switch by targeting EBV lytic genes.	[84]
EBV (LCLs)	PAR-CLIP	Identified 500 EBV miRNA targets.	Tested 29 miRNA:3’UTR combinations, identified by PAR-CLIP. There was >20% KD of luciferase expression for 21 out of the 29 PAR-CLIP-identified miRNA:3’UTR pairs.	p53 feedback loops, B cell activation, and apoptosis.	At least 14 EBV miRNAs, including those not encoded by B95-8, share seed sequence homology with cellular miRNAs.	[111]
KSHV (endothelial cells)	qCLASH	Identified 3324 target genes in 2 of 3 replicates, 1433 in 3 replicates.	Validated 30 of 54 identified miR-K11 targets.	Vascular endothelial growth factor (VEGF) pathway, apoptosis, cell cycle control, and glycolysis.	When looking at existing HITS-CLIP, 223 and 169 targets were shared with BC-1 and BC-3 cells, respectively.	[113]
EBV (Akata, SNU719)	qCLASH	Over 1700 viral and cellular targets.	In vivo validation—higher targeting efficacies of EBV miRNAs likely translate into stronger functional influences on their targets.	Antigen processing and presentation (MHC class I), ubiquitin and proteasome degradation, IFN-stimulated genes, and ISG15 antiviral mechanisms.	EBV miRNAs regulate the tumor cell phenotype and the immune cell microenvironment.	[110]

**Table 2:** ncRNAs and associated targets involved in antiviral immunity and oncogenesis.

Element (ncRNA/Protein)	Type	Origin	Immune Target(s)	Mechanism	Ref
ebv-miR-BART1-3p	miRNA	viral (EBV)	*IL12B*, miR-17, *CASP3* (Caspase 3)	Reduces CD4+ T cell differentiation, inhibits E2F3, decreasing miR-17, increases cell survival.	[45,49,72]
ebv-miR-BART1-3p	miRNA	viral (EBV)	miR-29a/b/c	Mimic, downregulated in NPC to increase cell migration/DNA methylation.	[45]
ebv-miR-BART1-5p	miRNA	viral (EBV)	LMP1, *IL12B*	Reduces EBV immunogenicity/evades immunity.	[49,50]
ebv-miR-BART2 -5p	miRNA	viral (EBV)	*IL12B*, *MICB*	Reduces CD4+ T cell differentiation, MHC I presentation.	[47,49]
ebv-miR-BART5-5p	miRNA	viral (EBV)	miR-18a/b	Mimic, upregulated in NPC; they are part of the oncogenic miR-17-92 cluster.	[45]
ebv-miR-BART5-3p	miRNA	viral (EBV)	IL2RB	Affects interleukin pathways	[117]
ebv-miR-BART6-5p	miRNA	viral (EBV)	Dicer, Rta, Zta, EBNA2	Establishment/maintenance of latency.	[68]
ebv-miR-BART6-3p	miRNA	viral (EBV)	RIG-I pathway, lncRNA-LOC553103	Downregulates IFN response (RIG-I genes), downregulates lncRNA-LOC553103, inhibits the metabolism and migration of tumor cells.	[56,158]
ebv-miR-BART8	miRNA	viral (EBV)	*STAT1*	Downregulates IFN response (IFN-γ-STAT1 signaling pathway).	[54]
ebv-miR-BART9-3p	miRNA	viral (EBV)	miR-141, -200a	Mimics, downregulates 141/200a to promote tumor.	[45]
ebv-miR-BART10-3p	miRNA	viral (EBV)	BHRF1	Increases BHRF1 protein levels and apoptosis.	[84]
ebv-miR-BART14-3p	miRNA	viral (EBV)	*IL2RB*	Affects interleukin pathways.	[117]
ebv-miR-BART15	miRNA	viral (EBV)	*NLRP3*	Restricts inflammasome activation.	[71]
ebv-miR-BART16	miRNA	viral (EBV)	LMP1, *CBP* (CREB binding protein), *TRIM8*, *CASP3*	Reduces EBV immunogenicity/evades immunity, downregulates IFN response, reduces antiviral immunity, increase cell survival.	[52,72,110,208]
ebv-miR-BART17-5p	miRNA	viral (EBV)	TAP2	CD8+ cell evasion.	[48]
ebv-miR-BART19-5p	miRNA	viral (EBV)	*DDX58* (RIG-1)	Decreases RIG-1 PRR expression.	[53]
ebv-miR-BART20-5p	miRNA	viral (EBV)	*TBX21*/T-bet, IFN- γ, BZLF1, BRLF1	Decreases cytotoxic cytokine production, downregulates IFN response (IFN-γ-STAT1 signaling pathway), promotes latency.	[54,70,208]
ebv-miR-BART22	miRNA	viral (EBV)	LMP2A	Decreases immunogenicity, promotes evasion/oncogenesis.	[209]
ebv-miR-BHRF1-2-3p	miRNA	viral (EBV)	*IL12B*	Reduces CD4+ T cell differentiation.	[49]
ebv-miR-BHRF1-2-5p	miRNA	viral (EBV)	*IL1R1*	Blocks IL-1 signaling.	[55]
ebv-miR-BHRF1-3	miRNA	viral (EBV)	*TAP2*, BZLF1	CD8+ cell evasion, suppresses lytic replication and gene expression.	[48,69]
hsa-miR-197	miRNA	host (EBV)	IL-6R	Upregulated in EBV BL, decreasing IL-6R.	[73]
circRPMS1_E4_E3a	lncRNA (circRNA)	viral (EBV)	unknown mechanism	Downregulates 11 cellular miRNAs, increased cell migration.	[142]
circLMP2A	lncRNA (circRNA)	viral (EBV)	miR-3908 (TDMD)	miR-3908/TRIM59/p53 axis. miR-3908 downregulated, TRIM59 upregulated, p53 degraded. Promotes invasion, metastasis and EMT	[151]
circBART2.2	lncRNA (circRNA)	viral (EBV)	RIG-I protein, IRF3	Activates PD-L1 and promotes tumor immune escape. Activates NF-κB, promotes tumor development.	[150]
circRELL1(4,5,6).1	lncRNA (circRNA)	host (KSHV) (EBV, HCMV)	TTI1	Causes pro-latency phenotypes via maintaining PI3K/AKT/mTOR pathway (KSHV). Also induced by EBV and HCMV.	[145,149]
circACADM(7,8,9,10).1	lncRNA (circRNA)	host (EBV)	miR-221-3p	miR-221-3p/CDKN1B axis for cell cycle dysregulation in EBV (increases miR-221, lowers CDKN1B).	[152]
circMDM2(6,7,8).1	lncRNA (circRNA)	host (EBV)	miR-589-5p	miR-589-5p/RPGR axis. Potential regulatory role in cancer.	[152]
circWDFY1(7,8,9).1	lncRNA (circRNA)	host (EBV)	no known mechanism	Upregulated in EBV NPC.	[152]
MALAT1	lncRNA	host (EBV)	miR-195	Sponges miR-195, increases PD-L1 and EBV tumor escape.	[157]
LMP1	protein	viral (EBV)	*H19*, *BIC*, miR-194, miR-146a	LMP1/H19/miR-675-5p/p53. Decreases H19 and miR-675-5p, increasing p53, promoting latency. Induces miR-155 activation in B-lymphocytes. Downregulates miR-194, which downregulates IL-10, and induces apoptosis. Induces miR-146a, inducing NF-κB.	[74,76,89,160,161]
EBNA3A, EBNA3C	protein	viral (EBV)	miR-222/221	Binds and activates miR-222/221.	[86]
EBER1	ncRNA	viral (EBV)	La protein IL-10	TLR3 activation, inflammation, activates RIG-1.	[176,177]
EBER2	ncRNA	viral (EBV)	PAX5	Promotes lytic infection, activates RIG-1.	[178]
miR-155	miRNA	host (KSHV/EBV)	NF-κB	Mimicked in KSHV. Activated in EBV.	[61,75,76,210,211]
miR-K12-1	miRNA	viral (KSHV)	NF-κB	Suppresses RTA to maintain latency.	[62]
kshv-miR-K12-3	miRNA	viral (KSHV)	nuclear factor I/B,C/EBPβ, hsa-miR-23	Suppresses lytic replication and gene expression; suppresses RTA to maintain latency. Increases IL-6 and IL-10 secretion. Hsa-miR-23 mimic.	[63,64,79]
kshv-miR-K12-5	miRNA	viral (KSHV)	*MYD88*	TLR/IL1-R signaling.	[58]
kshv-miR-K12-6-5p	miRNA	viral (KSHV)	miR-15/16	Mimics, inhibits cell cycle progression, decreases tumor	[80]
kshv-miR-K12-7-5p	miRNA	viral (KSHV)	*RTA*	Maintains latency.	[65]
kshv-miR-K12-7	miRNA	viral (KSHV)	MICB, C/EBPβ	MHC I. Increases IL-6 and IL-10 secretion.	[63]
kshv-miR-K12-9	miRNA	viral (KSHV)	IRAK1, RTA	TLR/IL1-R signaling. Maintains latency.	[58,66]
kshv-miR-K12-10a	miRNA	viral (KSHV)	miR-142-3p	Mimic of miR-142-3p	[79]
kshv-miR-K12-11	miRNA	viral (KSHV)	IKKɛ, miR-155	Maintains latency, controls IFN signaling (NF-κB), miR-155 mimic impacting B cell development.	[59,60]
circvIRF4	lncRNA (circRNA)	viral (KSHV)	Unknown	Potentially regulates gene expression.	[146]
miR-222/221	miRNA	host (KSHV)	p57^KIP2^	Downregulated in KSHV.	[85]
circHIPK3(2).1	lncRNA (circRNA)	host (KSHV)	miR-29b and miR-30c	ceRNA network circHIPK3(2).1/miR-29b/DLL4, to regulate cell cycle. Upregulated in KSHV (unknown how).	[147]
PAN	lncRNA	viral (KSHV)	JMJD3, UTX, PRC2	Regulates late gene expression, induces IL-4 level, decreases IFN-γ and IL-18 levels.	[123,124]
circPAN	lncRNA (circRNA)	viral (KSHV)	Unknown	Function not yet known.	[143,145]
vFLIP (K13)	protein	viral (KSHV)	miR-17, miR-146a. circSHROOM3(5).1, AL031123.1	Induces miR-17 to decrease TGF-β signaling pathway, promotes tumor. Downregulates CXCR4, upregulating miR-146a. circSHROOM3(5).1/hsa-miR-378i/SPEG/FOXQ1. Increase in miR-378i to inhibit KSHV reactivation. AL031123.1/hsa-miR-378i/SPEG/FOXQ1. increase in miR-378i to inhibit KSHV reactivation.	[90,164]
vIRF1	protein	viral (KSHV)	*LEF1*	Induces circARFGEF1(2,3,4).1, which degrades miR-125a-3p, inducing GLRX3, increasing cell proliferation and angiogenesis.	[148]

## Abbreviations

The following abbreviations are used in this manuscript:

EBV	Epstein–Barr virus
KSHV	Kaposi’s sarcoma herpesvirus
ncRNA	non-coding RNA
lncRNA	long non-coding RNA
NPC	nasopharyngeal carcinoma
EBVaGC	EBV-associated gastric carcinoma
DLBCL	diffuse large B cell lymphoma
KS	Kaposi’s Sarcoma
PEL	primary effusion lymphoma
MCD	multicentric Castleman’s disease
KICS	KSHV inflammatory cytokine syndrome
LANA	latency associated nuclear antigen protein
PRR	pattern recognition receptor
TLR	Toll-like receptor
MHC	major histocompatibility complex
NK	natural killer cells
IL	interleukins
IFN	interferon
ORF	open reading frame
BHRF1	BamHI fragment H rightward open reading frame 1
BART	BamHI fragment A rightward transcript
v-miRs	viral microRNAs
miRNA	microRNA
MICB	major histocompatibility complex class I-related chain B protein
NLRs	NOD-like receptors
LMP1	latent membrane protein
BZLF1	BamHI-Z leftward reading frame 1
RBP	RNA binding protein
GO	gene ontology
rLCV	rhesus lymphocryptovirus
RRV	rhesus rhadinovirus
EV, EVP	extracellular vesicle, extracellular vesicle and particle
AGO	Argonaute
RISC	RNA-induced silencing complex
MVB	multivesicular bodies
LECs	lymphatic endothelial cells
AGO-CLIP	Argonaute-cross-linking and immunoprecipitation
CLASH, qCLASH	Cross-Linking, Ligation and Sequencing of Hybrids, quantitative approach of CLASH
IL2RB	interleukin 2 receptor subunit beta
K-LEC	KSHV-infected lymphatic endothelial cell
ENE	expression and nuclear retention element
IRF	interferon regulatory factor
EBER	EBV encoded RNA
TR	terminal repeat
sisRNA	stable intronic sequence RNA
TMER	transfer RNA (tRNA)-miRNA encoding RNA molecules
circRNA	circular RNA
MHV68	murine gammaherpesvirus 68
lincRNAs	long intergenic non-coding RNAs
HCMV	human cytomegalovirus
ecircRNA	exonic circRNA
ciRNA	circular intronic RNA
EIcircRNA	exon-intron circRNAs
vIRF	viral interferon regulatory factor
PAN	long non-coding polyadenylated nuclear RNA
TDMD	target RNA-directed microRNA degradation
ceRNA	competing endogenous RNA
La protein	lupus erythematosus-associated antigen
Pre-miRNAs	miRNA precursors
HVS	herpesvirus saimiri
HHV	human herpesvirus
m6A	N^6^-methyladenosine RNA modification
m5C	5-methylcytosine RNA modification
Ac4C	N4-acetylcytidine modification
RLR	RIG-1-like receptor signaling
sORFs	short open reading frames
